# Seminal human papillomavirus originates from the body surface and is not a frequent aetiological factor in azoospermia

**DOI:** 10.1111/and.13202

**Published:** 2018-11-22

**Authors:** Jens Fedder, Dorthe Ørnskov, Birte Engvad, Thomas Kielsgaard Kristensen, Mads Lomholt, Niels Marcussen, Marianne Waldström

**Affiliations:** ^1^ Centre of Andrology & Fertility Clinic Odense University Hospital Odense C Denmark; ^2^ Give Hospital Friklinikken Give Denmark; ^3^ Department of Pathology Sygehus Lillebælt (Vejle) Vejle Denmark; ^4^ Department of Clinical Pathology Odense University Hospital Odense C Denmark

**Keywords:** azoospermia, human papillomavirus, male genital tract, semen, Vas deferens

## Abstract

Human papillomavirus (HPV) DNA has been detected in the testis tissue of 6.5% of 185 men with non‐obstructive azoospermia (NOA). Others have suggested that seminal HPV originates from contamination from the genital skin and mucosa. One hundred unselected azoospermic men and 43 normal men undergoing vasectomy were recruited. Testicular biopsies for HPV examination were collected from all the men. Additionally, the normal men undergoing vasectomy delivered a semen sample and had a swab for HPV examination taken from the genital skin before vasectomy. A piece of each Vas deferens obtained during the vasectomy was examined for the presence of HPV. Two of the primarily azoospermic men were shown to have cryptozoospermia. It was not possible to detect HPV in the testis tissue of any of the included 98 azoospermic men or the 43 proven fertile men. In the proven fertile men, HPV DNA was detected in the semen of 15 men (35%), on the genital skin of 28 men (65%), and in the Vas deferens in three cases (7%). In 13 (87%) men with HPV‐positive semen samples, HPV DNA was also detected in the skin swabs, and in 11 men (73%), identical HPV genotypes were found in the two locations.

## 1 INTRODUCTION

Azoospermia is found in 5%–20% of men suffering from infertility (Kolettis, 2002) and in 1%–2% of the overall population (Jarow, Espeland, & Lipschultz, 1989). Azoospermia can often be explained by genetic abnormalities (approximately 30%) or exogenic factors; however, in more than 20% of the cases, azoospermia remains unexplained (Fedder, Crüger, Østergaard, & Bruun Petersen, 2004).

Martorell et al. (2005) detected human papillomavirus (HPV) DNA in testicular biopsies from 12 (6.5%) of 185 men with non‐obstructive azoospermia (NOA). In this study, eight of the HPV‐positive men showed Sertoli‐cell‐only patterns by histological examination. The most frequent type, HPV 16, was detected in five cases (Martorell et al., 2005). Martorell et al. (2005) were not able to detect HPV in samples of testis tissue from 50 healthy men who died in traffic accidents. To the best of our knowledge, the study by Martorell et al. (2005) is the only previous study examining the presence of HPV in testis tissue, and it might suggest that HPV could be an aetiological factor in men in whom azoospermia until now has remained unexplained.

Human papillomavirus has also been found in the semen of infertile and healthy, fertile men in the range of ~2%–>30% (Laprise, Trottier, Monnier, Coutlée, & Mayrand, 2014). In a Danish study, we detected HPV in the spermatozoa from 16% of human sperm donors, and in 2/3 (10.6%), high‐risk types of HPV were detected (Kaspersen et al., 2011). Studies have suggested that seminal HPV originates from urogenital tract reservoirs (Damke et al., 2017) or from genital skin or mucosa (Giovannelli et al., 2007; Golob et al., 2014; Luttmer et al., 2015). Possible seminal “HPV contamination” from the genital skin and mucosa is supported by near associations between HPV types detected in the semen and on genital skin (Golob et al., 2014; Luttmer et al., 2015).

With the aim to highlight whether seminal HPV originates from a urogenital tract reservoir or is due to “contamination” from the genital mucosa and skin during ejaculation, we evaluated the following:

1. The prevalence of HPV in testicular tissue from azoospermic men compared to healthy, fertile men undergoing vasectomy, and
2. Associations between the presence of HPV in testicular tissue, the Vas deferens pieces removed by vasectomy, genital skin and semen.

## 2 MATERIALS AND METHODS

### 2.1 Study design and subjects

One hundred consecutive, unselected, non‐vasectomised, azoospermic men referred to the Centre of Andrology/Fertility Clinic, Odense University Hospital, were recruited. All non‐vasectomised azoospermic men referred to this centre are examined systematically as already described (Fedder, 2011; Fedder, Crüger, Østergaard, & Bruun Petersen, 2004). In short, history recording (incl. cryptorchidism, infections, medication and duration of infertility) and clinical examination including ultrasonography of the scrotum were performed. Men suggested to suffer from obstructive azoospermia furthermore had a rectal ultrasonography visualising the internal genitalia. Endocrinological analyses (incl. FSH, LH, testosterone, estradiol. prolactin, inhibin‐B, TSH and AMH) and genetic analyses (incl. karyotype, Y microdeletions and CFTR mutations) were in all cases performed. The presence of living, potential motile spermatozoa and testicular histology were in all cases evaluated by testicular biopsy as described below. In this study, in addition to routine procedures, testicular tissue from the biopsy was collected for HPV analyses, and for the final 35 included azoospermic men, genital skin swabs were taken for HPV analysis.

Forty‐three healthy, proven fertile men referred for vasectomy brought a fresh ejaculate on “the vasectomy day.” Swabs were taken before vasectomy as described below, and during the operation, a testicular biopsy was taken as described (Fedder, 2011).

The study was approved by the Scientific Ethics Committee of Southern Denmark (ID No: S‐20140189) and by the Data Protection Agency (No: 14/45374) and registered in ClinTrials.gov (NCT 02329275). All participants provided their written informed consent.

### 2.2 Collection of skin swabs

Before disinfection of the scrotal skin and vasectomy, a swab was taken from the entire penile surface and the anterior half of the scrotal surface using sterile gloves. A kit including a flocked nylon swab stick and a tube with 1 ml of transport medium was used. The swab was prewetted with the medium, and the swab stick was broken in the tube (Copan, Brescia, Italy); the screw cap was closed*, *and the sample was sent for analysis.

### 2.3 Collection and primary examination of ejaculates

When potential participants in a phone call accepted to consider participating, written information for participants and a plastic container for the ejaculate were sent by postal mail. On the day of vasectomy, the participants brought a relatively fresh ejaculate together with their informed consent. Since vasectomy might be stressful, some men preferred to produce the ejaculates in the evening before the operation. The ejaculates were in no cases older than 12 hr when delivered to the hospital, and they were all conserved in a plastic container in the temperature range between room temperature (20ºC) and body temperature (37ºC). However, because the interval between ejaculate production and analysis was often extended compared to intervals of <2 hr in routine infertility work‐up, only ejaculate volume and sperm concentration were read—using a Makler chamber (Sefi Medical Instruments Ltd., Haifa, Israel). Sperm motility was not analysed due to the variable and often several hours long periods from ejaculation to the delivery of the ejaculate.

### 2.4 Testicular biopsies

All testicular biopsies were performed after giving a funicular blockade with 200 mg of lidocaine (SAD Amgros I/S, København, Denmark) using a 14G TruCut needle (Aragon Medical Devices Inc., TX, USA) as described in detail by Fedder (2011). In men undergoing vasectomy, a testicular biopsy was always done unilaterally. When spermatozoa were found in our lab in the testicular tissue taken from the first testicle in men with azoospermia, we would take only a unilateral biopsy, unless bilateral biopsy was indicated due to anamnestic, clinical, or ultrasonographical findings (Fedder, 2011; Fedder, 2017). Testicular biopsies (cylindrical, 18 mm long [unless the testicle was smaller] and 1.3 mm in diameter [~24 mm^3^]) from all men were examined for HPV, and in addition, all azoospermic men were examined histologically for testicular pathology.

### 2.5 Examination of Vas deferens

During the vasectomy, ½–1 cm of each Vas deferens was routinely removed and sent for histological examination. The tissue was formalin‐fixed and paraffin‐embedded, and from the tissue block, three sections of 15 µm from each Vas deferens were cut. The sections from the right and left Vas deferens sections (right and left) from each man were mixed, and DNA was extracted as described below.

### 2.6 Detection of HPV DNA

DNA purification (of all samples) was performed using the Maxwell 16 LEV Blood DNA kit (AS1290; Promega Biotech, WI, USA). Pre‐treatment before DNA purification varied according to sample type. Three hundred microlitre of the swabs and the tissue slices of testis and Vas deferens samples were incubated with 330 µl Proteinase K suspension at 56°C for 1 hr and overnight, respectively, and thereafter used for DNA purification. From the ejaculates, 300 µl samples were incubated with 300 µl DTT (20 mM) for 30 min at 37°C. Thereafter, the samples were centrifuged, and the supernatant was discarded. The pellet was re‐suspended in 330 µl Proteinase K solution, incubated for 1 hr at 56°C and then used for DNA purification.

All samples were analysed for the presence of HPV using the INNO‐LiPA HPV assay (Fujirebio, Belgium) according to the manufactures protocol. In short, PCR was performed using biotinylated primers amplifying part of the L1‐region of the HPV genome (if HPV is present). A primer pair for the human gene HLA‐DPB1 was included in the PCR to ensure the presence and quality of the human sample. The PCR amplicons were denatured and bound to complementary DNA probes on a membrane. The presence of bound sample was visualised by the addition of streptavidin‐conjugated alkaline phosphatase and the BCIP/NBT chromogen.

The INNO‐LiPA HPV genotyping assay allowed us to identify the following HPV types: 6, 11, 16, 18, 26, 31, 33, 35, 39, 40, 42, 43, 44, 45, 51, 52, 53, 54, 56, 58, 59, 66, 68, 69, 70, 71, 73, 74 and 82.

### 2.7 Statistics

After controlling for normal distribution of the data, the ages of the azoospermic men and the healthy men for vasectomy were compared using Student's *t* test. The sperm concentrations, total sperm counts and ejaculate volumes in ejaculates from the HPV‐positive and HPV‐negative men undergoing vasectomy were compared using the Mann–Whitney test for comparison of unpaired data.

## 3 RESULTS

One hundred men referred for diagnostic testicular biopsy due to azoospermia had testicular biopsies examined for HPV DNA. During the diagnostic work‐up, including the examination of more than two ejaculates, two men (of the 100) were found to have cryptozoospermia with intermittent presence of spermatozoa in the ejaculates. The mean age of the azoospermic men was 33 years compared to 40 years for the healthy men undergoing vasectomy (*p* < 0.05).

Histological evaluations showed Sertoli‐cell‐only (SCO) syndrome in testicular biopsies from 38 men, hypoplasia/atrophia/maturation stop in 42 men and normal testicular tissue in 18 of the 98 men with permanent azoospermia (Table 1). Bilateral biopsy was performed in 26 (68%) of the 38 men in the SCO group, in 19 (45%) of the 42 men in the hypoplasia/maturation stop group and in only two (11%) of the 18 men with normal testicular histology. The 43 normal men undergoing vasectomy all had only unilateral testicular biopsy.

**Table 1:** Overview of aetiological factors detected in the 100 men primarily diagnosed with azoospermia shown for histological groups representing no sperm production, reduced sperm production or normal sperm production

	Total no. in the group	CFTR mutation (%)	Epididymitis antea (%)	Cryptorchidism (antea)(%)	Chromosome abnormality (%)	Y microdeletion (%)	Anabolic steroids	Chemo‐ and radiotherapy (%)	Other possible aetiologies (%)
Sertoli‐cell‐only syndrome	38	2 (~5)	1 (~3)	12 (~32)	1 (~3)	1 (~3)	1 (~3)	2 (~5)	3 (~8)
Hypoplasia/atrophia/maturation arrest	42	3 (~7)	1 (~2)	14 (~33)	1 (~2)	3 (~7)	2 (~5)	—	9 (~21)
Normal testis tissue	18	3 (~17)	8 (~44)	—	—	—	1 (~6)	—	5 (~28)
Cryptozoospermia	2		1 (~50)				1 (~50)		

Note

Participants belonging to specific aetiological groups are shown as numbers as well as percentages (in brackets) within each histological group.

The 18 men with normal testicular tissue had aetiologies characteristic of obstructive azoospermia (OA): 11 had Congenital Bilateral Absence of Vas deferens (CBAVD) based on a CFTR mutation or a history of epididymitis. For the azoospermic men with hypoplasia/atrophia/maturation arrest or SCO, the aetiologies represented a great spectrum. Twenty‐six (~33%) of the 80 patients had a history of cryptorchidism, and four (~5%) had a Y microdeletion. Other probable causes of azoospermia detected in the men with abnormal testicular tissue included chromosomal abnormalities (2), intake of anabolic steroids (3), chemotherapy (2), history of orchitis (1) and hypogonadotrophic hypogonadism (1). In only four (~5%) of the men with histologically abnormal testicular tissue, a CFTR mutation was detected, and one of these men in addition had a history of cryptorchidism and one a varicocele. Three of the 80 men had a history of severe genital chlamydia infections.

In the two men finally diagnosed as having cryptozoospermia, intratubular germinal cell neoplasia (IGCN) was detected in one case (who had been taking anabolic steroids 10 years earlier and had a history of a painful genital chlamydia infection treated with antibiotics), and a benign Leydig cell tumour in the other one. In the last case, the tumour was removed completely.

None of the 43 men recruited as proven fertile, healthy controls had a history of cryptorchidism or genital tract infections, and no abnormalities were noticed on the clinical routine screening before vasectomy. HPV DNA was detected in the semen of 15 men (35%), on the genital skin of 28 men (65%), in the Vas deferens in three cases (7%), and no testis tissue samples were HPV positive (Table 2). In 13 men (87%) with HPV‐positive semen samples, HPV was also detected on the skin swab, and in 11 men (73%), identical HPV types were found in the two locations (Table 2). The prevalence rates of HPV on the genital skin were ~69% among the subgroup of 35 of the azoospermic men examined.

**Table 2:** Detection of HPV on the genital skin, and in semen, Vas deferens*, *and Testis from the 43 healthy, proven fertile men undergoing vasectomy

Patient	Genital skin	Semen	Vas deferens	Testis
1	—	—	—	—
2	—	—	31	—
3	—	—	—	—
4	18	—	—	—
5	45	—	—	—
6	—	45	—	—
7	81	45, 53	—	—
8	16	16	—	—
9	—	—	—	—
10	51	—	—	—
11	44	—	—	—
12	—	—	—	—
13	61, 66	—	—	—
14	44	44	33, 59	—
15	—	—	—	—
16	18	—	—	—
17	45, 51, 68	45, 51, 68	—	—
18	51, 68	—	—	—
19	—	—	—	—
20	83	83	—	—
21	39	—	—	—
22	6, 11, 40, 51, 53, 66	6, 11, 39, 51, 53, 66	—	—
23	—	Unspec.	—	—
24	—	—	—	—
25	39	39	—	—
26	Unspec.	Unspec.	—	—
27	—	—	—	—
28	51	—	—	—
29	31, 53, 62, 73, 89	73	—	—
30	—	—	6	—
31	44	—	—	—
32	—	—	—	—
33	35	—	—	—
34	—	—	—	—
35	51	—	—	—
36	66	45, 66, 81	—	—
37	6, 11, 16, 18, 33, 40, 70	16, 33, 70	—	—
38	Unspec.	—	—	—
39	51	81	—	—
40	6	—	—	—
41	62	62	—	—
42	18, 66	—	—	—
43	—	—	—	—

No significant association between total sperm count, sperm concentration, ejaculate volume and presence of HPV in the semen or on the genital skin could be detected (Tables 3 and 4). The mean total sperm count for the 15 men with HPV in the semen was 196 mio/ejaculate compared to 300 mio/ejaculate in the 28 men without HPV in the semen (NS, Table 3).The mean total sperm count for the 28 men with HPV on the genital skin was 237 mio/ejaculate compared to 314 mio/ejaculate in the 15 men without HPV on the genital skin (NS, Table 4).

**Table 3:** Total sperm counts, sperm concentrations and ejaculate volumes (means ± *SD*'s followed by ranges in brackets) in the 43 proven fertile, healthy men mentioned according to presence and absence of HPV in the semen

	Seminal HPV detected	No seminal HPV detected	Significance level
Total sperm count (×10^6^)	196 ± 293 (2.2–1,099)	300 ± 309 (4.0–1,120)	*p* = 0.15 (NS)
Sperm concentration (×10^6^/ml)	71 ± 68 (5.5–190)	103 ± 94 (2.0–400)	*p* = 0.23 (NS)
Ejaculate volume (ml)	2.6 ± 1.9 (0.4–7.0)	2.9 ± 1.8 (0.2–8.0)	*p* = 0.38 (NS)

**Table 4:** Total sperm counts, sperm concentrations and ejaculate volumes (means ± *SD*'s followed by ranges in brackets) in the 43 proven fertile, healthy men mentioned according to presence and absence of HPV on the genital skin

	HPV detected on genital skin	No HPV detected on genital skin	Significance level
Total sperm count (×10^6^)	237 ± 313 (2.2–1,120)	314 ± 291 (4.0–1,040)	*p* = 0.33 (NS)
Sperm concentration (×10^6^/ml)	82 ± 78 (5.5–320)	111 ± 100 (2.0–400)	*p* = 0.23 (NS)
Ejaculate volume (ml)	2.6 ± 1.7 (0.4–7.0)	3.1 ± 2.0 (0.2–8.0)	*p* = 0.41 (NS)

## 4 DISCUSSION

It was not possible to detect HPV in the testis tissue from any of a heterogeneous group of 98 azoospermic men, two cryptozoospermic men or 43 proven fertile, healthy men undergoing vasectomy. However, HPV DNA was detected on the genital skin of 2/3 and in the semen in 1/3 of the healthy men. Due to a very high concordance of the specific HPV genotypes on the genital skin and in the semen, it may be suggested that HPV in the semen is due to contamination from the genital mucosa (and skin).

It might be difficult to recruit a control group of healthy volunteers undergoing a testicular biopsy. An important strength of this study is that the azoospermic men were matched with a control group of healthy, proven fertile men of similar age (40 versus 33 years). In addition, carefully systematic examination of the azoospermic men made it possible to compare the HPV status with testicular histology and aetiology of the azoospermia. Finally, the men undergoing vasectomy were examined for the presence of HPV DNA in four locations: unilateral testicular biopsy, the scrotal parts of the Vas deferens*,* the semen, and the penile and scrotal skin; this design serving as an appropriate basis to evaluate the natural history of HPV infection in the male genital tract. All samples from each man were collected on the same day.

The possible presence of HPV DNA was examined in an unselected cohort of azoospermic men with the purpose of comparing the prevalence in patients representing different aetiological groups. However, the chance to detect HPV in testicular tissue might have been increased by only including men with unexplained azoospermia (i.e., men without other detectable aetiological factors). It might also be considered a weakness that only one HPV test was included for the detection of HPV DNA. However, the INNO‐LiPA HPV assay is a very widely used assay that is considered sensitive and validated and detects 32 of the most important HPV types (Else et al., 2011; van Hamont, Ham, Bakkers, Massuger, & Melchers, 2006).

Human papillomavirus was previously detected in 6.5% of 185 men with NOA (Martorell et al., 2005). However, we were not able to detect HPV in testicular biopsies from neither 43 normal men nor 98 azoospermic men representing a wide spectrum of aetiologies including both NOA and OA.

Human papillomavirus DNA can be found in semen from infertile and normal, healthy men. A recent systematic review including 27 studies demonstrated a mean prevalence of HPV DNA of 16% (95% confidence intervals [CI]: 10%–23%) in men from infertile couples and 10% (95% CI: 7%–14%) in men from other populations (Laprise et al., 2014). This large variation might be due to the inclusion of many small heterogenic studies. The prevalence of HPV depends upon ethnicity, numbers of sexual partners, anal sex with other men and circumcision (Nielson et al., 2007). Additionally, in semen donors, HPV DNA prevalence rates of 7.5%–26.3% have been detected (Kaspersen et al., 2013, 2011). A contributing explanation of the high prevalence of HPV DNA in the semen of 35% of 43 proven fertile men found in this study might be the modest size of this control group.

A slightly, but not significantly, lower sperm concentration/total sperm count observed in this study was in agreement with the literature (Gizzo et al., 2014). A few studies have demonstrated an association between seminal HPV DNA analysed by Linear Array HPV and Fluorescence in Situ Hybridization (FISH) and sperm motility (Foresta et al., 2010; Lai et al., 1997). However, in other studies, it has not been possible to demonstrate an association between the presence of HPV in the semen and sperm motility (Golob et al., 2014). Since it was not always possible for the healthy volunteers in this study to deliver the ejaculates within our usual time limits of 1–2 hr, we did not systematically evaluate sperm motility.

Human papillomavirus DNA in semen might originate from a male genital tract reservoir or could be a result of contamination of HPV from the skin/mucosa during ejaculation. In agreement with the results of Golob et al. (2014) and Luttmer et al. (2015), the control group in this study showed a high prevalence of HPV DNA on genital skin swabs and in semen, and the concordance of HPV genotypes detected in the two locations was high.

These prevalence rates of more than 60% having HPV on the genital skin as found in this study are higher than the prevalence rates of ~37% found in infertile Slovenian men referred for fertility treatment (Golob et al., 2014) and ~34% healthy male volunteers from the Netherlands (Luttmer et al., 2015). The higher prevalence rates found in this study might be explained by geographical differences. We took swabs from the anterior scrotum and all over the penile shaft but not under the foreskin. Conversely, Luttmer et al. (2015) included swabbing from the glans, coronal sulcus, frenulum and inner part of the foreskin when taking penile scrapes. In Golob et al. (2014), the swabs from the penile surface were taken by self‐swabbing after careful instruction, including hand washing before masturbation.

Similarly, the prevalence of ~35% fertile men having HPV in the semen in this study was higher than a prevalence of ~14% found in infertile men in Golob et al. (2014) and ~16%–27% found by Luttmer et al. (2015) depending on whether figures were based on examinations of 1, 2 or 3 ejaculates. A higher prevalence of ~35% having HPV in semen compared to a prevalence of ~16% found in the semen from Danish sperm donors (Kaspersen et al., 2011) might be explained by a slightly increased age in the men having vasectomy compared to the younger semen donors.

Human papillomavirus DNA in the Vas deferens has been detected in at least two studies. Rintala, Pöllänen, Nikkanen, Grénman, and Syrjänen (2002) found HPV DNA in the Vas deferens of five (18.5%) of 27 healthy men undergoing vasectomy. Additionally, HPV DNA was found in five (27.8%) seminal plasma samples delivered by 18 of the 27 men at 6 months after vasectomy. Absolutely, no correlation between the presence of HPV types in the Vas deferens and postoperatively obtained seminal plasma could be detected, and seminal plasma HPV was suggested to originate from genital tract reservoirs in the prostate or the seminal vesicles (Rintala et al., 2002). Based on the most recent knowledge, it is obvious to suggest that the HPV DNA in the postoperatively obtained seminal fluid is due to contamination of HPV from the skin or mucosa.

In another study by Svec, Mikysková, Hes, and Tachezy (2003), HPV DNA was detected in the epididymis in six of 22 men with a histologically confirmed diagnosis of non‐tuberculous epididymitis. In addition, Vas deferens tissue available from five of the 22 patients was positive for HPV DNA in one case (Svec et al., 2003), revealing a prevalence of HPV DNA close to the 3/43 (~7%) found in this anatomical location in the present study.

Since HPV is known to cause malignancy in the female Cervix uteri and in the anus, penis and pharynx (Garolla et al., 2012), it is reasonable to ask whether HPV is a cause of testicular cancer. Garolla et al. (2012) found a higher prevalence of HPV in the semen of 155 testicular cancer patients compared to controls. They observed that patients who received radio‐ and/or chemotherapy had significantly higher HPV prevalence rates compared to untreated patients (Garolla et al., 2012). The increased prevalence after treatment might be due to the decreased clearance of the HPV infections because the immune system might have been inhibited by the cancer treatment. However, with examination of human seminoma tissue from 61 patients and normal testicular tissue from 23 men, Bertazzoni et al. (2013) was not able to detect HPV DNA in anyone.

The majority of sexually active people will acquire at least one HPV infection during their lifetime (Dunne, Nielson, Stone, Markowitz, & Giuliano, 2006; Nielson et al., 2007). However, more than 90% of these HPV infections are cleared by the immune system within months or a few years from the primary exposure (Woodman, Collins, & Young, 2007). Clearance depends upon the HPV type and anatomical localisation.

Foresta et al. (2015) found that HPV DNA clearance in the semen improved after vaccination with the quadrivalent HPV vaccine Gardasil (Merck Serono S.p.A., Milan, Italy). If HPV in the semen is due to contamination from the mucosa during ejaculation, these data might reflect clearance of HPV from the body surface (mucosa and skin).

### 4.1 The future

In the majority of men with azoospermia, a probable cause can be identified (Fedder et al., 2004; Fedder, 2017). Since it was not possible to detect HPV DNA in the testicular biopsies from unselected azoospermic men in the present study, HPV is probably not a frequent cause of azoospermia. To highlight the aetiologies of azoospermia, and particularly NOA not explained by other probable causes, it might be relevant to evaluate other factors than HPV, for example possible genetic factors (Stouffs et al., 2016) including the expression of microRNA (Noveski et al., 2016).

## CONFLICT OF INTEREST

The authors declare no conflict of interest.

## AUTHORS' CONTRIBUTIONS

JF designed the study in collaboration with the other authors. JF informed all participating and non‐participating potential study participants, collected the written informed consent, performed all genital skin swabs and testicular biopsies, and collected and analysed all ejaculates from the men undergoing vasectomy. DØ performed all HPV analyses, and BE and NE carried out histological evaluations of testicular tissues from all azoospermic and cryptozoospermic men. ML performed the vasectomies. All authors contributed to the final article based on a draft written by JF.
